# Optical spin-orbit interaction induced by magnetic textures

**DOI:** 10.1038/s41598-026-52576-7

**Published:** 2026-07-09

**Authors:** Martin Luttmann, Mauro Fanciulli, Pietro Carrara, Maurizio Sacchi, Thierry Ruchon

**Affiliations:** 1 Université Paris-Saclay, CEA, LIDYL, Gif-sur-Yvette, 91191 France; 2 DQML, IMX, École Polytechnique Fédérale de Lausanne (EPFL), Station 12, CH-1015 Lausanne, Switzerland; 3 CY Cergy Paris Université, CEA, LIDYL, Gif-sur-Yvette, 91191 France; 4 New Technologies Research Center, University of West Bohemia, Plzeň, 30100 Czech Republic; 5 Sorbonne Université, CNRS, Institut des NanoSciences de Paris, INSP, F-75005 Paris, France

**Keywords:** Magneto-optics, Circular dichroism

## Abstract

Contrary to the optical spin angular momentum (SAM), the role played by the orbital angular momentum (OAM) of light in magneto-optics remains largely unexplored. However, the SAM and OAM are known to be coupled when light interacts with non-homogeneous and non-isotropic materials. Here we predict that the OAM carried by each photon in a light beam is modified upon reflection on magnetic textures like skyrmions, and that the sign and magnitude of this variation are governed both by the SAM of the incident field and by the topology of the texture. Our predictions can be readily tested by performing circular dichroism measurements, and may provide new routes to read the topology of magnetization optically, or to shape light’s angular momentum with magnetism.

## Introduction

Spin-orbit interaction (SOI) is a phenomenon occurring in many areas of quantum physics, for instance in condensed matter in the case of noncollinear magnetism^1^, magnetocrystalline anisotropy^2^, Rashba^3^ and Dresselhaus effects^4^, anomalous Hall effect^5^, spin Hall effects^6^, topological materials^7^ and the electronic structure of heavy element compounds such as transition metal dichalcogenides^8^, or in dilute matter for gas phase spectroscopy^9^. More recently, this concept was extended to the spin and orbital angular momenta (SAM and OAM) of light, opening the field of *optical spin-orbit interactions* (OSOI). Through OSOI, light angular momentum of one type – spin or orbital – may be controlled by the value of the other type^10^; in this respect, OSOI is a quite distinct concept from electronic SOI in condensed-matter systems. OSOI has been identified upon strong focusing conditions, or through refraction or diffraction. However, OSOI was also reported when light propagates in a medium that is both anisotropic and inhomogeneous, with important applied perspectives for the control of the SAM and OAM of light. This is the working principle of optical elements like q-plates^11,12^, whichexhibit spatially varying birefringence), metasurfaces^13,14^ or light-shaping nanostructures^15,16^. These examples all rely on a structural patterning of matter, possibly controlled by electric fields. On the contrary, while the coupling between light polarization and magnetism is well established^17–21^, the research effort aiming at identifying the role of OAM in magneto-optical interactions is only very recent^22–26^ and studies of OSOI induced by magnetism are very scarce today^27,28^. However, as the ensemble of stable magnetic structures, with fine control on their manufacturing and innumerable applications, is growing, it is timely to investigate their specific impact on light beams carrying SAM and OAM. Some promising textures like magnetic vortices and skyrmions^29–33^ are particularly attractive for these investigations. They are patterned in dots of swirling magnetization, while their typical size ranges from a few microns, to a few nanometers, which is comparable to focal spot sizes of visible to soft X-ray beams, a spectral range where light beams with angular momentum are now available^34,35^. In this context, identifying magnetically-induced OSOIs might open interesting perspectives and new avenues for technological applications. These effects may be used as new platforms for probing non-trivial magnetic topologies, similarly to the techniques using vortex beams for chiral sensing^36,37^. The ultrafast control of the angular momentum of light^38,39^ may also benefit from such a study.

Here we harness the theoretical framework proposed in Ref^40^. to demonstrate how the average OAM per photon in a light beam can be modified upon reflection on magnetic textures. In particular, we show that the sign of the OAM variation is controlled by the SAM of the incident field, while its magnitude depends on the specific magnetic structure considered. This magnetically-induced OSOI originates from the azimuthal interference between the longitudinal, transverse and polar magneto-optical Kerr effect (MOKE) components, that get coupled for incident elliptically polarized light fields. Taking the example of the X-ray spectral range, we show that magnetic vortices and skyrmions are an ideal test-bed for the observation of this form of OSOI.

## Results

### Magnetic OSOI

The notations introduced in Ref^40^. will be used throughout this paper, with simplifications when possible; we will note *ℓ* the topological charge of the beam (corresponding to the average OAM), and *s* the SAM of the beam. For a pure mode of OAM *ℓ* and SAM *s*, they correspond to the OAM and SAM of individual photons divided by *ℏ*. We consider an incident vortex beam^41–43^ that is a pure mode of OAM quantum number $\ell _{\text {in}}$. It writes, in the (*p*, *s*) polarization basis, as

1 $$\begin{aligned} \vec {E}_{\text {in}}=A(r,z)e^{-i\ell _{\text {in}}\phi }\begin{pmatrix} \epsilon _p\\ \epsilon _s \end{pmatrix}\,. \end{aligned}$$

*A*(*r*, *z*) is a scalar function, with radial (*r*) and longitudinal (*z*) dependence, which can for instance correspond to a Laguerre-Gaussian (LG) amplitude^41^. *ϕ* is the azimuthal angle, as measured with respect to the center of the beam. $(\epsilon _p, \epsilon _s)$ is a unit vector describing the polarization state of the incident field, (1, 0) and (0, 1) corresponding to *p*-polarization and *s*-polarization, respectively. Circular polarization is expressed as $(\epsilon _p, \epsilon _s) = \frac{1}{\sqrt{2}}(1, -i\,s_{\text {in}})$, $s_{\text {in}} = +1$ and *-1* corresponding respectively to circular right (CR) and circular left (CL) polarization. The incident beam is reflected by a planar magnetic sample with three-dimensional magnetization. Considering the linear MOKE, the reflected field $\vec {E}_{\text {out}}$ is given by the product of $\vec {E}_{\text {in}}$ with the reflectivity matrix^19,44^

2 $$\begin{aligned} \textbf{R}=\begin{pmatrix} r_{pp}\cdot \left[ 1+ r_0^t \cdot m^t\right] & r_{ps}^l \cdot m^l+ r_{ps}^p\cdot m^p \\ - r_{ps}^l\cdot m^l+ r_{ps}^p\cdot m^p & r_{ss} \end{pmatrix}\,, \end{aligned}$$

where $m^t$, $m^l$, $m^p$ are the transverse, longitudinal and polar components of the magnetization with respect to the plane of incidence, normalized by the saturation magnetization. $r_{pp}$ and $r_{ss}$ are the standard Fresnel reflectivity coefficients and $r_0^t$, $r_{ps}^l$ and $r_{ps}^p$ are magneto-optical constants. They depend on the material, the wavelength and the incidence angle^19,45^. As we consider an inhomogeneous sample, it should be noted that this reflectivity matrix generally depends on both *r* and *ϕ*.

To simplify the analytical derivations, as in Ref^40^., we assume a close to normal incidence, so that we can identify the coordinates in the transverse plane of the beam and in the sample plane. Moreover, we drop the radial dependence of the magnetization of simplicity, as it is not expected to have an impact on the OAM content. Note that we will use in our analytical calculations the magneto-optical constants computed at Brewster’s angle. Comparing to realistic simulation results far from normal incidence in the following section, we will confirm the soundness of these approximations to capture the essence of magnetically-induced OSOI. Since the magnetization must be *2π* periodic with *ϕ*, its components can be expressed as azimuthal Fourier series^40^

3 $$\begin{aligned} m^\dag (\phi ) = m_0\sum _{n=-\infty }^{+\infty }a_n^\dag e^{in\phi }\,, \end{aligned}$$

where the *\dag = t,l,p* can refer to each of the three components of the magnetization, and where $m_0$ is a dimensionless quantity. The field after reflection off the magnetized material writes, using Eq. 1, 2 and 3,

4 $$\begin{aligned} \vec {E}_{\text {out}}&= {\textbf {R}} \cdot \vec {E}_{\text {in}}\nonumber \\&=A(r,z)e^{-i\ell _{\text {in}}\phi } \begin{pmatrix} \epsilon _pr_{pp}\cdot \left[ 1+ r_0^t m^t\right] + \epsilon _s\cdot \left[ r_{ps}^l m^l + r_{ps}^p m^p \right] \\ \epsilon _p\cdot \left[ -r_{ps}^l m^l + r_{ps}^p m^p \right] +\epsilon _s r_{ss} \end{pmatrix}\nonumber \\&=A(r,z) \begin{pmatrix} \epsilon _p r_{pp} e^{-i\ell _{\text {in}}\phi } + m_0 \sum _n\left[ \epsilon _p r_{pp}r_0^t a_n^t + \epsilon _s\left( r_{ps}^l a_n^l + r_{ps}^p a_n^p \right) \right] e^{i(-\ell _{\text {in}}+n) \phi } \\ \epsilon _s r_{ss} e^{-i\ell _{\text {in}}\phi } + m_0 \sum _n \epsilon _p \left( -r_{ps}^l a_n^l + r_{ps}^p a_n^p \right) e^{i(-\ell _{\text {in}}+n) \phi } \end{pmatrix}\,. \end{aligned}$$

For a general incident elliptical polarization state, terms corresponding to transverse, longitudinal and polar MOKE contribute to the *p*-polarized component of the reflected field, while the *s*-polarized component has contributions from longitudinal and polar MOKE only. We also notice that the OAM $\ell _{\text {in}}$ of the incident field has been redistributed over the OAM modes $\ell _{\text {in}}+n$, the weight of each mode being proportional to $\left| \epsilon _p r_{pp}r_0^t a_n^t + \epsilon _s\left( r_{ps}^l a_n^l + r_{ps}^p a_n^p \right) \right| ^2 + \left| \epsilon _p \left( -r_{ps}^l a_n^l + r_{ps}^p a_n^p \right) \right| ^2$, for *n ≠ 0*. From Eq. 4 we see that a uniform magnetization (i.e. the $a_0^{\dag }$ terms) contributes, as expected, to mixing the *p* and *s* components, while it does not contribute to OAM redistribution nor to OSOI. As we are interested in the variation of OAM, we set $a_0^\dag = 0$ in what follows, namely we only consider inhomogeneous magnetization patterns. We denote respectively $\mathcal {W}_{\text {in}}$ and $\mathcal {W}_{\text {out}}$ the energies in the incoming and outgoing beams. It can be shown that (see Eq. S42 of the supplementary material)

5 $$\begin{aligned} \mathcal {W}_{\text {out}} =\mathcal {W}_{\text {in}}\cdot \left[ \left| \epsilon _p r_{pp}\right| ^2 + \left| \epsilon _s r_{ss}\right| ^2 + |m_0|^2 \sum _{n \ne 0} \left( \left| \epsilon _p r_{pp}r_0^t a_n^t +\epsilon _s \left( r_{ps}^l a_n^l + r_{ps}^p a_n^p \right) \right| ^2 + \left| \epsilon _p \left( -r_{ps}^l a_n^l + r_{ps}^p a_n^p \right) \right| ^2 \right) \right] \,. \end{aligned}$$

The mean OAM quantum number for the reflected field is (Eq. S43 of the supplementary material)

6 $$\begin{aligned} \ell _{\text {out}} = \ell _{\text {in}} + |m_0|^2\cdot \frac{\mathcal {W}_{\text {in}}}{\mathcal {W}_{\text {out}}} \cdot \sum _{n \ne 0} n \left( \left| \epsilon _p r_{pp}r_0^t a_n^t + \epsilon _s \left( r_{ps}^l a_n^l + r_{ps}^p a_n^p \right) \right| ^2 + \left| \epsilon _p(-r_{ps}^l a_n^l + r_{ps}^p a_n^p)\right| ^2 \right) \,. \end{aligned}$$

Similarly, the mean SAM quantum number in the outgoing beam, projected on the propagation axis, reads (Eq. S45)

7 $$\begin{aligned} {\begin{matrix} s_{\text {out}} & =2\frac{\mathcal {W}_{\text {in}}}{\mathcal {W}_{\text {out}}}\cdot \left[ \mathcal {I}\left( \epsilon _p^*\epsilon _s r_{pp}^* r_{ss} \right) + |m_0|^2\cdot \sum _{n\ne 0}{\mathcal {I}\bigg (\left[ \epsilon _p r_{pp} r_0^t a_n^t + \epsilon _s \left( r_{ps}^l a_n^l+r_{ps}^p a_n^p\right) \right] ^*\cdot \epsilon _p \left( -r_{ps}^l a_n^l+r_{ps}^p a_n^p\right) \bigg )} \right] \,. \\ \end{matrix}} \end{aligned}$$

At this point, several conclusions can been drawn. First, in the case of a homogeneous magnetization, which corresponds to $a_n^\dag =0$ for *n≠ 0*, we see that there is no change of OAM, as anticipated from Eq.4. Second, for an inhomogeneous magnetization we find that neither the OAM nor the SAM changes depend on the magnetization direction, i.e. on the sign of $m_0$, but rather on its modulus square and on the relative weights of its Fourier components. Third, the OAM of the incoming field does not affect the SAM of the outgoing field (Eq. 7), while the opposite may not be true (Eq. 6). Indeed, as we will show later, a non-null incident SAM will cause an OAM variation. We identify this OAM change, controlled by the SAM, to a magneto-optical SOI. Finally, we conclude from Eq. 6,7 that in general the variation of OAM is not compensated by an opposite variation of SAM. This is expected, as a non-zero incidence angle as well as a spatially varying magnetization may both break the rotational symmetry of the problem.

The formulae given in Eq. 5, 6 and 7 greatly simplify in cases of high rotational symmetry, such as magnetic vortices or skyrmions. In the following calculations, we will consider a planar magnetic vortex of order $N_{\text {Sk}}$^40^, which approximates a Bloch skyrmion. In this case, there is no azimuthal variation of $m^p$, and the azimuthal variations of $m^t$ and $m^l$ are purely harmonic, with periodicity *2π*; we thus have $a_n^p=0$, and only the $n=\pm N_{\text {Sk}}$ terms contribute to $a_n^t$ and $a_n^l$:

8 $$\begin{aligned} {\begin{matrix} a_n^t & = {\left\{ \begin{array}{ll} \frac{1}{2}, \, n = \pm N_{\text {Sk}} \\ 0, \, n \ne \pm N_{\text {Sk}} \end{array}\right. }\\ a_n^l & = {\left\{ \begin{array}{ll} \pm \frac{i}{2}, \, n = \mp N_{\text {Sk}} \\ 0, \, n \ne \pm N_{\text {Sk}} \end{array}\right. }\\ a_n^p & = 0 \end{matrix}} \,. \end{aligned}$$

Note that a more realistic skyrmion texture, having a non-zero polar component, will be used for the simulations in the following section. Here, the reflected beam is in a superposition of modes $\ell _{\text {in}}$, $\ell _{\text {in}}+N_{\text {Sk}}$ and $\ell _{\text {in}}-N_{\text {Sk}}$ and the change in OAM reduces to

9 $$\begin{aligned} \Delta \ell = \ell _{\text {out}} - \ell _{\text {in}}&=N_{\text {Sk}}|m_0|^2\frac{\mathcal {W}_{\text {in}}}{4\mathcal {W}_{\text {out}}}\cdot \bigg ( \left| \epsilon _p r_{pp}r_0^t + i\epsilon _sr_{ps}^l\right| ^2 - \big .\nonumber \big .\left| \epsilon _p r_{pp}r_0^t - i\epsilon _sr_{ps}^l\right| ^2 \bigg ) \nonumber \\&=N_{\text {Sk}}|m_0|^2\frac{\mathcal {W}_{\text {in}}}{\mathcal {W}_{\text {out}}}\cdot \mathcal {I} \bigg ( \epsilon _p \epsilon _s^* \cdot r_{pp}r_0^t \cdot r_{ps}^{l,*} \bigg )\,. \end{aligned}$$

**Fig. 1:**
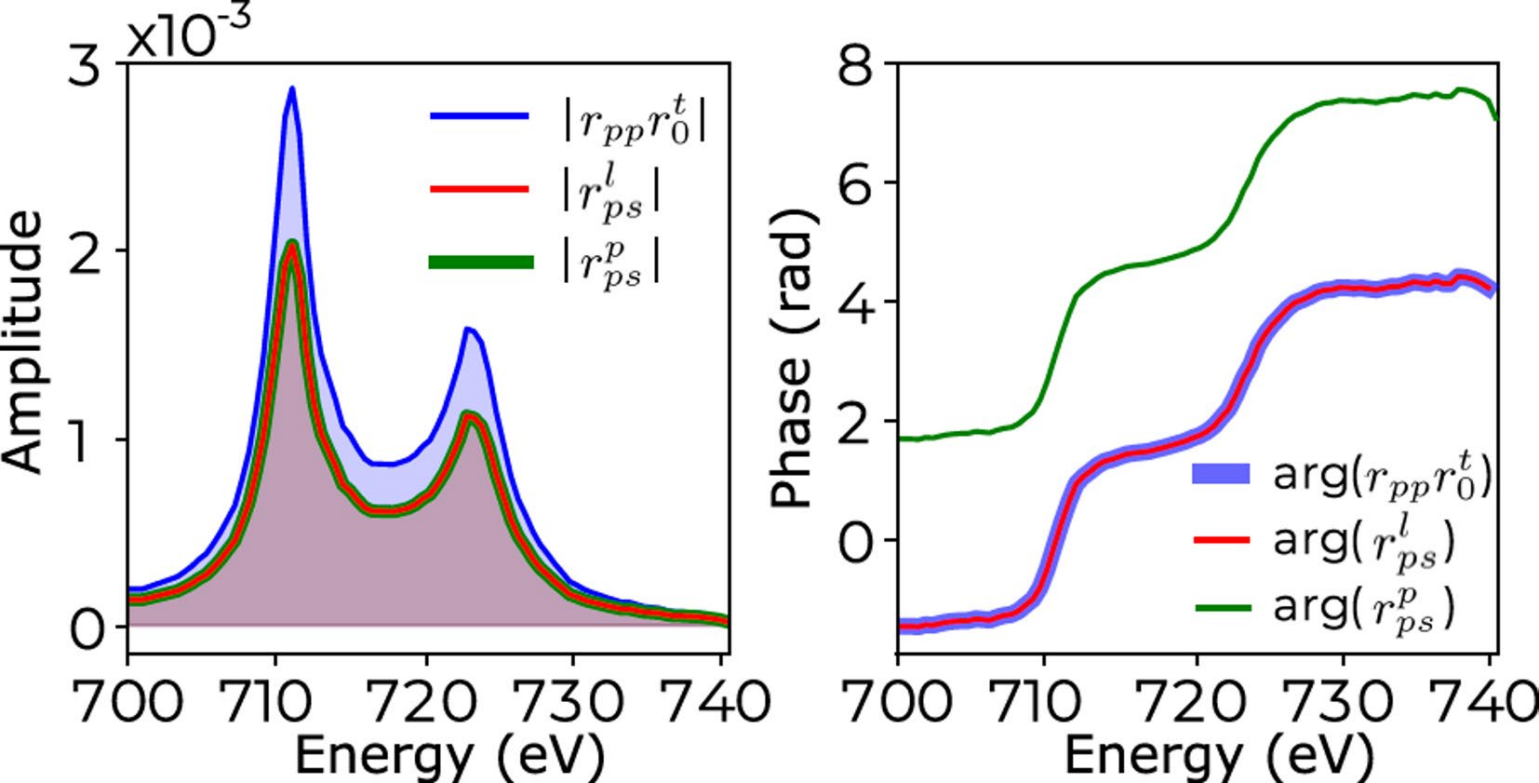
Amplitude (left) and phase (right) of the magneto-optical constants $r_{pp}r_0^t$, $r_{ps}^l$ and $r_{ps}^p$ at the L-edge of iron, for an angle of incidence of 45°.

In practice, $r_{pp}r_0^t$ and $r_{ps}^l$ constants may often have the same phase. As a practical example, we consider the L$_3$-edge of Fe, corresponding to a photon energy of 711 eV, and a wavelength of about 1.7 nm (see Section I of the supplementary material for the derivation of the constants). Figure 1 shows the different magneto-optical constants at Brewster’s angle, with respect to the photon energy. We observe that the three constants have similar amplitudes. We also note that $r_{pp}r_0^t$ and $r_{ps}^l$ have the exact same complex phase, but are *π* out of phase with $r_{ps}^p$. Even though our calculations were conducted assuming normal incidence, in the following we take into account the magneto-optical constants at Brewster’s incidence, where they have the largest amplitude. Indeed, the objective of our analytical calculation is to identify the relevant parameters of the OSOI effect, rather than obtaining exact results. The relevance of the formulae obtained will be validated with realistic simulations in the next section of this paper. Further details about this procedure may be found in Ref^25^.. Thus, in the region of the L$_3$-edge of Fe, we finally get

10 $$\begin{aligned} \Delta \ell =N_{\text {Sk}}|m_0|^2 \frac{\mathcal {W}_{\text {in}}}{\mathcal {W}_{\text {out}}}\cdot \mathcal {I} \big (\epsilon _p\epsilon _s^* \big )\cdot \left| r_{pp}r_0^t r_{ps}^{l,*}\right| \,. \end{aligned}$$

At this point, we can differentiate two cases, according to the incident polarization.

**Fig. 2:**
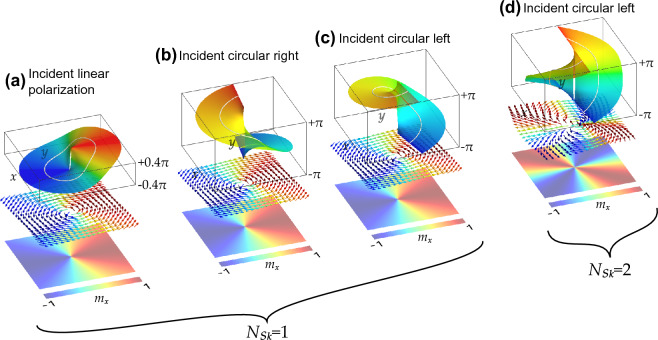
Spatial dephasing of the *p* component of the field upon reflection off a vortex of swirling magnetization. The isosurface color indicates the local phase of the reflected beam, while the bottom images show the transverse magnetization magnitude $m_x$. (**a**) For an incident *p*-polarized field, the variation of OAM is null, as the net dephasing along a loop about the optical axis (white line) is null. (**b**,**c**) For an incident CR ($s_{\text {in}}=1$) and CL ($s_{\text {in}}=-1$) field. There is a *2π* dephasing along the loop, thus a positive (**b**) or negative (**c**) OAM variation. Higher-order vortices induce higher variations of the OAM (**d**). Here we obtain a *4π* azimuthal phase variation with a magnetic vortex of order $N_{\text {Sk}}=2$.

If the incident light is linearly polarized, which corresponds to $\epsilon _p$ and $\epsilon _s$ having the same phase, one finds $\ell _{\text {out}} = \ell _{\text {in}}$. Figure 2.a shows the kick of transverse phase of the *p* component of the light upon reflection [i.e. the phase of $\epsilon _p(r_{pp} + r_{pp}r_0^t m^t) + \epsilon _s(r_{ps}^l m^l + r_{ps}^p m^p)$], for a planar magnetic vortex and a *p*-polarized incident field. While the magneto-optical interaction does induce a curvature of the beam wavefront, the mean OAM is unchanged, as the accumulated phase along a loop surrounding the optical axis is zero.

If the incident light is circularly polarized, we find

11 $$\begin{aligned} \Delta \ell = s_{\text {in}}N_{\text {Sk}} \frac{\mathcal {W}_{\text {in}}}{\mathcal {W}_{\text {out}}}\cdot \frac{ |m_0|^2}{2}\left| r_{pp}r_0^t r_{ps}^l\right| \,. \end{aligned}$$

We thus unveil a form of OSOI, since the OAM change can be positive or negative depending on the helicity of the incident field $s_{\text {in}}$ and the value of the magnetic winding number $N_\text {Sk}$ (Fig. 2.b,c), and cancels out if one of the two is equal to zero. Returning to Eq. 4, we note that this change of OAM only occurs in the *p*-polarized component of the beam, whereas the *s*-polarized component retains an OAM equal to $\ell _{\text {in}}$. Indeed, the *p* component of the reflected field contains the interference between the transverse and longitudinal magnetization terms, coupled through the presence of both $\epsilon _p$ and $\epsilon _s$, resulting in a variation of OAM. On the other hand, the *s* component of the reflected field is simply given by a term proportional to the longitudinal magnetization, up to a constant term. Note however that in the presence of azimuthally-varying longitudinal *and* polar magnetization components, an OAM variation can also occur in the *s* component of the light field. We can also compute the mean outgoing SAM quantum number as

12 $$\begin{aligned} s_{\text {out}}&=2 \frac{\mathcal {W}_{\text {in}}}{\mathcal {W}_{\text {out}}}\left[ \mathcal {I}\left( \epsilon _p^*\epsilon _s r_{pp}^*r_{ss} \right) - \frac{|m_0|^2}{2} \mathcal {I}\left( \epsilon _p \epsilon _s^*\right) \left| r_{ps}^l\right| ^2 \right] \nonumber \\&=\frac{\mathcal {W}_{\text {in}}}{\mathcal {W}_{\text {out}}}\left[ -s_{\text {in}} \mathcal {R}\left( r_{pp}^*r_{ss} \right) + s_{\text {in}} \frac{|m_0|^2}{2} \left| r_{ps}^l\right| ^2 \right] \,. \end{aligned}$$

The first term on the right hand side is the well-known change of polarization upon reflection on a surface due to the dephasing between the *s* and *p* polarization components. For instance, setting $r_{pp}=-r_{ss}=1$, which would model a perfect metallic mirror at 45$^{\circ }$ incidence, we retrieve the expected inversion of SAM. We notice the strong analogy between the second term and the OAM change in Eq. 11, which are similar up to the replacement of one $\left| r_{ps}^l\right|$ term by $\left| r_{pp}r_0^t\right|$. However, the difference in magnitude prevents them from compensating one another, and we conclude that the total angular momentum of light is not conserved during the interaction. This results from the fact that the sample is not at a normal angle with respect to the incident beam’s propagation axis, and thus does not show rotation symmetry about this axis. This is again analog to non-rotationally symmetric q-plates, which do not allow a pure SAM to OAM conversion^11^.

### Simulation of the magneto-optical interaction

To test our predictions, we perform simulations of a realistic MOKE experiment, not at normal incidence, using the magneto-optical constants at the L$_3$-edge of Fe. The photon energy of the incident beam is 711.2 eV, and the incidence angle is 45$^\circ$ (Brewster’s angle). We express the spin texture of the magnetic skyrmions using the following ansatz^46,47^:

13 $$\begin{aligned} {\begin{matrix} {\textbf {m}}_{\text {Sk}} & = (m^l, m^t, m^p)\\ & = \bigg ( C(\phi ) \sin \{f(r)\}, S(\phi ) \sin \{f(r)\}, \cos \{f(r)\} \bigg ) \,, \end{matrix}} \end{aligned}$$

with $C(\phi )=\cos (N_{\text {Sk}}\phi +h)$ and $S(\phi )=\sin (N_{\text {Sk}}\phi +h)$. The helicity *h* determines the type of the skyrmion. *h = (0, π )* corresponds to Néel-type solutions (with zero azimuthal magnetization component) and $h =\pm \pi /2$ yields Bloch-type solutions (a magnetization vortex). $N_{\text {Sk}}$ is the skyrmion charge, equal to 1 for the Bloch and Néel skyrmions considered here. We will later comment on the case of textures with $N_{\text {Sk}}>1$. A good approximation of the radial function *f*(*r*) is^47^

14 $$\begin{aligned} f(r) =4 \tan ^{-1}\big (e^{-\frac{r}{\lambda }} \big ) \,, \end{aligned}$$

where *λ* is the extension of the skyrmion. In numerical applications, we will set *λ = 100*nm, in order to match the size of typical skyrmions. The incident beam waist is also set to 100 nm. The other parameters of the simulation (in particular the exact modelling of magnetic sample and substrate) are the same as in Ref^25,40^.. The reflected field at the sample’s plane (Eq. 4) is numerically propagated to the far field using the Fraunhofer operator.

**Fig. 3:**
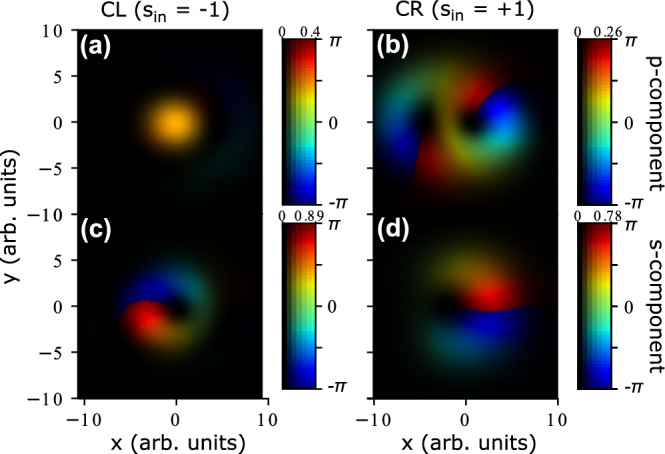
Analysis of the reflected beam in the far field, for a CL (left column) and CR (right column) incident field with $\ell _{\text {in}}=1$, impinging on an order 1 Bloch skyrmion. (**a**,**b**) *p*-polarization component. (**c**,**d**) *s*-polarization component. The phase is indicated by the color, and the local intensity corresponds to the brightness of the image.

Figure 3 displays the results for an incident LG mode of OAM $\ell _{\text {in}}=1$, with $s_{\text {in}}=\pm 1$, impinging on a Bloch skyrmion. For $s_{\text {in}}=-1$, inspecting the intensity and phase of the *p* and *s* components of the reflected field at the detector (Fig. 3.a,c), we observe that the *p* component has been almost fully converted into the *ℓ =0* mode, as it exhibits a strong on-axis intensity and flat spatial phase. As expected, the *s* component retains the *ℓ =1* helical phase of the incident beam. On the other hand, for $s_{\text {in}}=+1$ an increase in OAM is manifest in the phase of the *p* component, which shows a *4π* azimuthal variation, typical of an OAM of 2 (Fig. 2.b). Similar observations can be made for a Néel-type skyrmion (Fig. S1 of the supplementary material). The reflected field is a superposition of different modes, and thus does not exhibit the perflectly round, donut-like profile typical of pure LG modes.

**Fig. 4:**
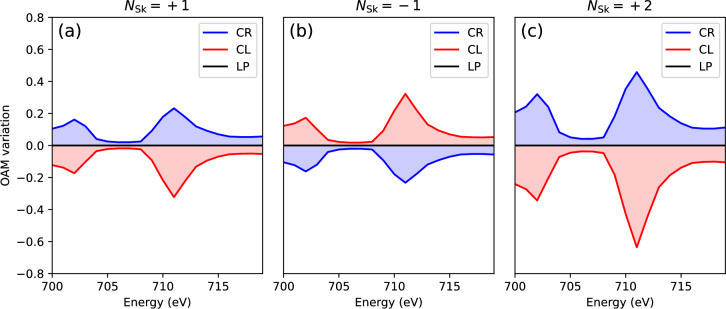
OAM variation per photon $\Delta \ell$ upon reflection on a Bloch skyrmion of helicity *h=π /2*, with respect to the photon energy at Brewster’s angle, for (**a**) $N_{\text {Sk}}=1$, (**b**) $N_{\text {Sk}}=-1$ and (**c**) $N_{\text {Sk}}=2$, for incident CR (blue), CL (red) and LP (black) polarization.

To get a more quantitative insight of the OAM variation, we compute numerically the local OAM value in the far field using Eq. S24 (see Fig.S1.e-h). This quantity is then weighted by the local intensity and averaged over the beam profile, yielding the average OAM value per photon in the reflected beam. Subtracting the incident OAM $\ell _{\text {in}}$, we obtain the mean OAM variation $\Delta \ell = \ell _{\text {out}} - \ell _{\text {in}}$ caused by the reflection on the skyrmion. $\Delta \ell$ is shown as a function of the photon energy for various winding number $N_{\text {Sk}}$ in Fig. 4. As expected from the analytical calculations, we find that in the vicinity of the L-edge of Fe, around 710 eV, $\Delta \ell$ is positive for a CR incident polarization ($s_{\text {in}} =+1$) and negative for a CL incident polarization ($s_{\text {out}} =-1$). In particular, at the resonance frequency, we find $\Delta \ell \approx \pm 0.3$ for $N_{\text {Sk}}=1$ and $\Delta \ell \approx \pm 0.6$ for $N_{\text {Sk}}=2$ (corresponding to a higher order skymrion^33^). Finally, the sign of $\Delta \ell$ changes with the sign of $N_{\text {Sk}}$. This is in full agreement with our approximate, analytical calculations. We notice a slight asymmetry between the CR and CL curves. Although we have not entirely identified its origin so far, we believe it may be due to the magnetic circular dichroism caused by the polar magnetization component of the skyrmions. Finally, for completeness, the curve of $\Delta \ell$ with respect to the incidence angle is available in Fig S2 of the supplementary material.

### On-axis magnetic circular dichroism

**Fig. 5:**
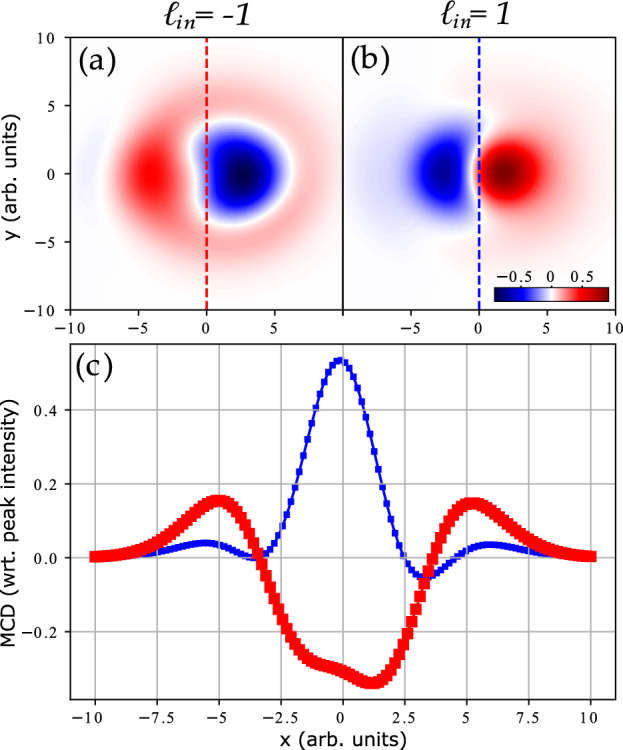
Magnetic circular dichroism images for a *h= π /2* Bloch skyrmions (top images), and for an incident OAM $\ell _{\text {in}}=-1$ (**a**, **b**) and $\ell _{\text {in}}=1$ (**c**,**d**). (**e**) Line-outs of the MCD images along the dashed lines of corresponding colors in (**a**-**d**). The dichroic signal is given as a fraction of the peak intensity.

For a $\ell _{\text {in}} = 1$ beam incident on a skyrmion, the reflected beam may contain a *ℓ =0* contribution, depending on the incident SAM. Inspecting Fig. 3.a, we observe that the absence of optical singularity in the *p* component leads to a Gaussian-like on-axis intensity profile, whereas the presence of an optical singularity results in a very weak on-axis intensity (Fig. 3.b). Thus, a MCD measurement, consisting in subtracting the far field images obtained with $s_{\text {in}}=1$ and $s_{\text {in}}=-1$, performed with a $\left| \ell _{\text {in}}\right| =1$ incident OAM, should exhibit a measurable on-axis signal. Figure 5 shows the predicted dichroic images, for $\ell _{\text {in}}=\pm 1$ incident OAM.

The 2D integral of these images is zero. Yet, as predicted, they exhibit an on-axis positive signal for $\ell _{\text {in}}=1$ and negative signal for $\ell _{\text {in}}=-1$ incident polarization (see the line-outs in Fig. 5.c), independently of the magnetic skyrmion helicity, as the OSOI is independent of the sign of $m_0$ (Eq. 11). The peak amplitude of the signal is strong: at maximum about 0.93 times the peak intensity of the reflected beam, and between 30% and 50% on the optical axis. The fact that the strongest signal is not observed on axis is due to the reflected beam being a non-pure sum of LG modes, as mentioned in the previous section. MCD images thus provide an experimental observable of OSOI. Contrary to the magnetic helicoidal dichroism images reported in Ref^25^., here the OSOI signature is not contained in the overall shape of the image, but in its net value - positive or negative - in a localized region of space.

## Discussion

We identified a form of OSOI occurring in magneto-optics with structured magnetic samples. Our study focused on reflection off a magnetic interface, described by the MOKE formalism. This effect arises from the interference between the transverse, longitudinal and polar MOKE effects, coupled by the electric field’s components of the incident light. We have shown that the helicity of the incident beam determines the OAM content and the average OAM quantum number of the reflected field. Remarkably, the magnitude and sign of the OAM variation is also governed by the winding number of the magnetic texture, and cancels out for a winding number equal to zero. This indicates that this effect could be used to implement all-optical ultrafast skyrmion readers, which would encode the magnetic state on light’s spatial degrees of freedom. OAM demultiplexer could then be used to decode the information. In this paper, we numerically computed the effect for X-ray light, but it is expected to exist at all wavelengths, including in the visible, to the cost of a reduced magnitude.

This modification of the OAM of the light is not accompanied by an opposite change of the SAM, that would yield a null total angular momentum modification. Therefore, part of light’s angular momentum is coherently transferred to matter. How this transfer exactly takes place in our case – is a mechanical torque exerted on the texture, perhaps via the excitation of spin waves? – will have to be investigated in future works. Conversely, the Faraday effect^17^, wherein the incident beam is transmitted by a magnetic sample, should allow to observe full SAM to OAM transfer, since, in that case, the sample could be invariant under rotation about the beam axis.

We argue that an experimental setup similar to the one reported in Refs^25,26^ could be used to observe this OSOI effect by performing MCD measurements. Our work allows to anticipate that magnetic textures with appropriate topology could serve as re-configurable OAM beam shapers, similarly to q-plates and metasurfaces in the visible domain, or patterned spintronic emitters in the Terahertz range^39^. Compared to these passive devices, magnetic textures present the promising advantage of being controllable by external magnetic or electric fields, and exhibit a fast dynamical response. One could also imagine shaping or controlling magnetic skyrmions with beams of light carrying SAM and/or OAM^23,48,49^, with applications in data encoding and processing^33^. Finally, the capability of free-electron lasers^50^ or high harmonic generation sources^34,35,51,52^ to synthesise ultrashort femtosecond or attosecond light pulses carrying various kinds of angular momentum lets us envision to perform the MCD experiment described here at the natural time-scales of spin dynamics in matter.

## Supplementary Information

Supplementary Information.

## Data Availability

The simulation data is available upon reasonable request to the authors of the paper.
