# Correction: Optical spin-orbit interaction induced by magnetic textures

**DOI:** 10.1038/s41598-026-65311-z

**Published:** 2026-08-04

**Authors:** Martin Luttmann, Mauro Fanciulli, Pietro Carrara, Maurizio Sacchi, Thierry Ruchon

**Affiliations:** 1 Université Paris-Saclay, CEA, LIDYL, 91191 Gif-sur-Yvette, France; 2 DQML, IMX, École Polytechnique Fédérale de Lausanne (EPFL), Station 12, CH-1015 Lausanne, Switzerland; 3 CY Cergy Paris Université, CEA, LIDYL, 91191 Gifsur-Yvette, France; 4 New Technologies Research Center, University of West Bohemia, Plzeň, 30100 Czech Republic; 5 Sorbonne Université, CNRS, Institut des NanoSciences de Paris, INSP, F-75005 Paris, France

Correction to: *Scientific Reports* 10.1038/s41598-026-52576-7, published online 09 July 2026

The original version of this Article contained an error in the name of author Thierry Ruchon which was incorrectly given as Thierrry Ruchon.

The original Article has been corrected.

