# Supplementary material for: Optical spin-orbit interaction induced by magnetic textures
Source: Sci Rep. 2026 Jul 9;16:21422. doi: 10.1038/s41598-026-52576-7 (PMC13350730; doi:10.1038/s41598-026-52576-7)
Supplement: Supplementary file 1 — Supplementary Information. [file 41598_2026_52576_MOESM1_ESM.pdf]

SUPPLEMENTARY MATERIALS : Optical spin-orbit interaction induced by magnetic textures

Martin Luttmann,[1, 2, *] Mauro Fanciulli,[3, 1, 4] Pietro Carrara,[5] Maurizio Sacchi,[5] and Thierry Ruchon[1, †]

[1] *Université Paris-Saclay, CEA, LIDYL, 91191 Gif-sur-Yvette, France*
[2] *DQML, IMX, Ecole Polytechnique Fédérale de Lausanne (EPFL) Station 12, CH-1015 Lausanne, Switzerland*
[3] *CY Cergy Paris Université, CEA, LIDYL, 91191 Gif-sur-Yvette, France*
[4] *New Technologies Research Center, University of West Bohemia, 30100 Plzeň, Czech Republic*
[5] *Sorbonne Université, CNRS, Institut des NanoSciences de Paris, INSP, F-75005 Paris, France*
(Dated: April 29, 2026)

I. MAGNETO-OPTICAL CONSTANTS

The values of the magneto-optical constants [53] are computed from available Faraday effect measurements in the spectral region of interest [54], using established formulas [45, 55]. These formulas also use the complex refractive index, that we extract from the Center for X-Rays Optics database [56].

II. MODAL DECOMPOSITION AND PHYSICAL OBSERVABLES OF OPTICAL BEAMS

In this appendix, we establish the expression of energy, linear and angular momenta of a beam decomposed on a Laguerre-Gaussian basis or equivalent. After recalling the general definitions given in Ref. [57], we first apply them to beams in a single mode, before generalizing to multimodal beams of interest in the main text.

A. General definitions

All through, we consider a monochromatic field, and denote respectively $\vec{E}$ and $\vec{H}$ its associated electric and magnetic fields, where for conciseness, we drop the space and time explicit dependence. The quantities of interest are, in Gaussian units :

- The time-averaged energy density:

$$W = \frac{g\omega}{2}\left(|\vec{E}|^2 + |\vec{H}|^2\right) \tag{S1}$$

where ω if light's angular frequency, and $g = 1/(8\pi\omega)$.

- The (canonical) momentum density:

$$\vec{P} = \frac{g}{2}\mathcal{I}\left[\vec{E}^* \cdot (\nabla)\,\vec{E} + \vec{H}^* \cdot (\nabla)\,\vec{H}\right] \tag{S2}$$

with

$$\vec{E}^* \cdot (\nabla)\,\vec{E} = \begin{pmatrix} \sum_j E_j^* \frac{\partial E_j}{\partial x} \\ \sum_j E_j^* \frac{\partial E_j}{\partial y} \\ \sum_j E_j^* \frac{\partial E_j}{\partial z} \end{pmatrix}. \tag{S3}$$

where$j = x, y, z$. Here $\mathcal{I}$ stands for the imaginary part. An analog formula could be written for $\vec{H}$.

* Correspondence email address: martin.luttmann@epfl.ch
† Correspondence email address: thierry.ruchon@cea.fr

- The orbital angular momentum density:

$$\vec{L} = \vec{r} \times \vec{P} \tag{S4}$$

- The spin angular momentum density:

$$\vec{S} = \frac{g}{2}\mathcal{I}\left[\vec{E}^* \times \vec{E} + \vec{H}^* \times \vec{H}\right] \tag{S5}$$

We will denote $\mathcal{W}$, $\mathcal{P}$, $\mathcal{L}$ and $\mathcal{S}$, the corresponding quantities integrated over the transverse spatial coordinates, which are respectively identified with the total energy, linear momentum, OAM and SAM carried by the beam.

B. A single monochromatic mode of any polarization and given OAM

To give a first example, we consider the following field in the paraxial approximation with both an elliptical polarization and a given helical phase, corresponding to a single OAM:

$$\vec{E} = A(\vec{r}, z)e^{ikz+i\ell\phi}\begin{pmatrix}\epsilon_p \\ \epsilon_s \\ 0\end{pmatrix} \; ; \; \vec{H} = \vec{\nabla} \times \vec{E} \simeq \vec{e}_z \times \vec{E}, \tag{S6}$$

with $|\epsilon_p|^2 + |\epsilon_s|^2 = 1$. Note that the longitudinal fields (electric and magnetic) are neglected as a consequence of the paraxial approximation. We dropped the harmonic time dependence, $-\omega t$, and consider that $A(\vec{r}, z)$ is a slowly varying envelope as compared to kz. With Eq. S1, we get immediately the time averaged energy density

$$W = g\omega \left|A(r, z)\right|^2 \tag{S7}$$

For evaluating (S2), we first consider the electric field. It has no z-component, so the sums in (S3) run over the first two indices only. The derivatives for the z-components are of the form $\partial E_j/\partial z$. If we neglect the envelope derivatives, it reads ikE_j and the sum for this z-component is $\sum_j E_j^* ikE_j = ik|A(r, z)|^2$. The x, y components require derivatives of the form $\partial E_j/\partial x$ or $\partial E_j/\partial y$. With the change of coordinates

$$x = r\cos\phi \tag{S8}$$

$$y = r\sin\phi \tag{S9}$$

and using the corresponding Jacobi matrix we have

$$\frac{\partial}{\partial x} = \cos\phi\frac{\partial}{\partial r} - \frac{\sin\phi}{r}\cdot\frac{\partial}{\partial\phi} \tag{S10}$$

$$\frac{\partial}{\partial y} = \sin\phi\frac{\partial}{\partial r} + \frac{\cos\phi}{r}\cdot\frac{\partial}{\partial\phi} \tag{S11}$$

In the following, we will discard the radial derivative, considering the beam's envelope much larger than the region of interest. The derivative of any component E_j against x (resp. y) thus reads $-i\ell\frac{\sin\phi}{r}\cdot E_j$ (resp. $i\ell\frac{\cos\phi}{r}\cdot E_j$). We thus have

$$\begin{aligned}
E_x^*\frac{\partial E_x}{\partial x} + E_y^*\frac{\partial E_y}{\partial x} &= -i\ell\frac{\sin\phi}{r}|A(r, z)|^2 \\
E_x^*\frac{\partial E_x}{\partial y} + E_y^*\frac{\partial E_y}{\partial y} &= i\ell\frac{\cos\phi}{r}|A(r, z)|^2 \\
E_x^*\frac{\partial E_x}{\partial z} + E_y^*\frac{\partial E_y}{\partial z} &= ik|A(r, z)|^2
\end{aligned} \tag{S12}$$

and

$$\frac{g}{2}\mathcal{I}\left[\vec{E}^* \cdot (\nabla)\,\vec{E}\right] = \frac{g}{2}|A(r, z)|^2\begin{pmatrix}-\ell\frac{\sin\phi}{r} \\ \ell\frac{\cos\phi}{r} \\ k\end{pmatrix} \tag{S13}$$

For the magnetic part, we get

$$\begin{aligned}\frac{g}{2}\cdot\mathcal{I}\left[\vec{H}^*\cdot(\nabla)\vec{H}\right] &= \frac{g}{2}\cdot\mathcal{I}\left[\vec{e}_z\times\vec{E}^*\cdot(\nabla)\vec{e}_z\times\vec{E}\right]\\ &= \frac{g}{2}\cdot\mathcal{I}\left[\begin{pmatrix}-E_y^*\\ E_x^*\\ 0\end{pmatrix}\cdot(\nabla)\begin{pmatrix}-E_y\\ E_x\\ 0\end{pmatrix}\right].\end{aligned}$$

It is just the same as before, but exchanging the x and y components, and a sign change on E_y. Inspecting (S12), we realize that it is just the same contribution. We thus retrieve the result given in Ref. [57]: the time-averaged density of linear momentum reads

$$\vec{P} = g|A(r,z)|^2\begin{pmatrix}-\ell\frac{\sin\phi}{r}\\ \ell\frac{\cos\phi}{r}\\ k\end{pmatrix} = \frac{W}{\hbar\omega}\left(\frac{\ell\hbar}{r}\vec{e}_\phi + \hbar k\vec{e}_z\right) \tag{S14}$$

where $\vec{e}_\phi$ (resp. $\vec{e}_z$) is the azimuthal (resp. longitudinal) unit vector. It is the density of photons ($\frac{W}{\hbar\omega}$) times the local linear momentum of a photon, which, in addition to the common contribution $\hbar k\vec{e}_z$, includes a second term depending on the tilt of the wavefront through $\frac{\ell\hbar}{r}\vec{e}_\phi$.

The time-averaged local OAM density then writes

$$\vec{L} = \vec{r}\times\vec{P} = \frac{W}{\hbar\omega}\left(\ell\hbar\vec{e}_z - k\hbar r\vec{e}_\phi\right) \tag{S15}$$

It is the density of photons times the projection of the OAM of each photon on the propagation direction ($\ell\hbar$), plus an azimuthal circulating OAM with a null average.

For the SAM time-averaged density, we get, using Eq. S7

$$\begin{aligned}\vec{S} &= \frac{g}{2}|A(r,t)|^2\mathcal{I}\left[\begin{pmatrix}0\\ 0\\ \epsilon_p^*\epsilon_s - \epsilon_s^*\epsilon_p\end{pmatrix} + \begin{pmatrix}-\epsilon_s^*\\ \epsilon_p^*\\ 0\end{pmatrix}\times\begin{pmatrix}-\epsilon_s\\ \epsilon_p\\ 0\end{pmatrix}\right]\\ &= 2g|A(r,t)|^2\mathcal{I}(\epsilon_p^*\epsilon_s)\cdot\vec{e}_z\\ &= \frac{2W}{\hbar\omega}\mathcal{I}(\epsilon_p^*\epsilon_s)\cdot\hbar\vec{e}_z\end{aligned} \tag{S16}$$

We note, as expected, that for $(\epsilon_p,\epsilon_s) = \frac{1}{\sqrt{2}}(1,-si)$, with $s=\pm1$, i.e. a circularly polarized beam, the expression reduces to $\vec{S} = \frac{W}{\hbar\omega}\cdot s\hbar\vec{e}_z$; i.e. the density of photons times $s\hbar\vec{e}_z$, the SAM per photon.

C. A multimodal beam

We now consider a coherent superposition of several such components, involving each a single OAM and a given polarization. The total field is written

$$\vec{E} = \sum_n A_n(r,z)e^{ikz+i\ell_n\phi}\begin{pmatrix}\epsilon_{p,n}\\ \epsilon_{s,n}\\ 0\end{pmatrix}\ ;\ \vec{H}\simeq\vec{e}_z\times\vec{E}, \tag{S17}$$

with

$$(\epsilon_{p,n})^2 + (\epsilon_{s,n})^2 = 1 \tag{S18}$$

The expansion of Eq.S18 only involves the index ℓ_n; a possible radial index of Laguerre-Gauss modes is absorbed in the amplitude $A_n(r,z)$. For further transverse spatial averaging we will use

$$\int d\phi e^{i(\ell_n-\ell_{n'})\phi} = 2\pi\delta_{n,n'}, \tag{S19}$$

and define

$$|c_n(z)|^2 = \int 2\pi r dr|A_n(r,z)|^2. \tag{S20}$$

Note the longitudinal dependence of $c_n(z)$. In the case of a laser pulse, it would serve encoding the temporal profile of the nth component.

All products of the previous section will result in double sums, i.e. interference terms. We do not detail the case of $\vec{H}$, anticipated to double all results. For instance, for the energy density we get

$$W(r,\phi,z) = 2\frac{g\omega}{2} \cdot \sum_{n,n'} A_n(r) A^*_{n'}(r) \cdot \left(\epsilon_{p,n}\epsilon^*_{p,n'} + \epsilon_{s,n}\epsilon^*_{s,n'}\right) e^{i(\ell_n - \ell_{n'})\phi} \tag{S21}$$

The linear density of energy in the beam is obtained by integrating over the transverse coordinates r, and ϕ. Using Eqs. S19 and Eq. S20 we get

$$\mathcal{W}_{out}(z) = g\omega \cdot \sum_n |c_n(z)|^2 \tag{S22}$$

One more integration along z would yield the total energy of the light pulse. In the following, we consider that all components have the same temporal shape. Up to a constant, which depends on this specific temporal shape, the linear density of energy can be identified to the total energy of the pulse and we will therefore drop the z-dependence of $\mathcal{W}_{out}$. The same considerations hold for all linear densities below.

For the linear momentum density $\vec{P}$, the procedure is very similar. For the electric part, we have (Eq. S2)

$$\begin{aligned}\vec{P}_E &= \frac{g}{2}\mathcal{I}\left[\vec{E}^* \cdot (\nabla)\, \vec{E}\right] \\ &= \frac{g}{2} \cdot \mathcal{I}\left[\sum_{n,n'} A^*_n(r) A_{n'}(r) \cdot \left(\epsilon^*_{p,n}\epsilon_{p,n'} + \epsilon^*_{s,n}\epsilon_{s,n'}\right) \cdot \begin{pmatrix} -i\ell_{n'}\frac{\sin\phi}{r} \\ i\ell_{n'}\frac{\cos\phi}{r} \\ ik \end{pmatrix} e^{i(\ell_{n'} - \ell_n)\phi}\right]\end{aligned} \tag{S23}$$

The magnetic part will give an identical contribution (the same argument as in the previous section is valid for the global field), giving

$$\vec{P} = g \cdot \mathcal{I}\left[\sum_{n,n'} A^*_n(r) A_{n'}(r) \cdot \left(\epsilon^*_{p,n}\epsilon_{p,n'} + \epsilon^*_{s,n}\epsilon_{s,n'}\right) \cdot e^{i(-\ell_n + \ell_{n'})\phi}\left(\frac{i\ell_{n'}}{r}\vec{e}_\phi + ik\vec{e}_z\right)\right]$$

The integration over the azimuth of the longitudinal part (along $\vec{e}_z$) will select $n = n'$. For the term proportional to $\vec{e}_\phi$, the situation is different. For instance, the x-component has a phase term $\left(e^{i\phi} - e^{-i\phi}\right) \cdot e^{i(\ell_n - \ell_{n'})\phi}$. So, only terms with $\Delta\ell = \pm 1$ will survive. The same is observed for the y-component. We do not calculate it any further, but the energy flow gets structured, with a momentum spinning around $\vec{e}_z$.

Now, we can compute the local time-averaged OAM density as

$$\vec{L} = \vec{r} \times \vec{P} \tag{S24}$$

$$= g \cdot \mathcal{I}\left[\sum_{n,n'} A^*_n(r) A_{n'}(r) \cdot \left(\epsilon^*_{p,n}\epsilon_{p,n'} + \epsilon^*_{s,n}\epsilon_{s,n'}\right) \cdot e^{i(-\ell_n + \ell_{n'})\phi}\left(i\ell_{n'}\vec{e}_z - ikr\vec{e}_\phi\right)\right] \tag{S25}$$

Its projection along the propagation axis, integrated over the transverse space (r,ϕ) reads, taking into account Eqs. S19, Eq. S20 and Eq. S22

$$\mathcal{L}_{out,z} = g \cdot \sum_n \ell_n |c_n|^2 \tag{S26}$$

$$= \frac{\mathcal{W}_{out}}{\hbar\omega}\frac{\sum_n \ell_n |c_n|^2}{\sum_n |c_n|^2}\hbar. \tag{S27}$$

We note that the polarization of each mode is not playing any role; the OAM linear density is just the sum of the $\ell_n \hbar$ terms for each mode, multiplied by their weights and the total number of photons.

For the spin angular momentum density, we proceed the same way. For the electric part, we have

$$\vec{S}_E = \frac{g}{2}\mathcal{I}\left[\vec{E}^* \times \vec{E}\right] \tag{S28}$$

$$= g \cdot \mathcal{I}\Bigg[\sum_{n,n'} A_n(r)^* A_{n'}(r) \cdot \left(\epsilon_{p,n}^* \epsilon_{s,n'} - \epsilon_{s,n}^* \epsilon_{p,n'}\right) \cdot e^{i(\ell_{n'}-\ell_n)\phi} \cdot \vec{e}_z\Bigg] \tag{S29}$$

The magnetic part will gives an equal contribution. Upon integration over both r and the azimuth, ϕ, only the $n = n'$ terms survive. Noting that $\mathcal{I}\left(\epsilon_{p,n}^* \epsilon_{s,n'} - \epsilon_{s,n}^* \epsilon_{p,n'}\right) = 2\mathcal{I}(\epsilon_{p,n}^* \epsilon_{s,n})$ we get

$$\mathcal{S}_{out} = 2g \cdot \sum_n |c_n|^2 \mathcal{I}(\epsilon_{p,n}^* \epsilon_{s,n}) \tag{S30}$$

$$= 2\frac{\mathcal{W}_{out}}{\hbar\omega} \cdot \frac{\sum_n |c_n|^2 \mathcal{I}(\epsilon_{p,n}^* \epsilon_{s,n})}{\sum_n |c_n|^2} \cdot \hbar \vec{e}_z. \tag{S31}$$

The SAM density is directed along the propagation axis z, and simply corresponds to the weighted sum of the modes respective SAMs multiplied by the number of photons.

D. Application to magnetically induced SOI

To match Eq. 4 of the main text with Eq. S17, we set

$$\ell_n = \ell_{in} + n \qquad \forall n. \tag{S32}$$

We denote

$$\mathcal{W}_{in} = g\omega \int 2\pi r dr |A(r,z)|^2; \tag{S33}$$

a quantity proportional to the total energy of the incoming field. As above, skipping the temporal shape dependence of the proportionality constants, we will directly identify it with the energy in the incoming beam. We also set, skipping again the z-dependence, for $n = 0$:

$$A_0(r) = \left[\left|\epsilon_p r_{pp} \cdot \left[1 + m_0 r_0^t a_0^t\right] + \epsilon_s m_0 \left[r_{ps}^l a_0^l + r_{ps}^p a_0^p\right]\right|^2 + \left|\epsilon_s r_{ss} + \epsilon_p m_0 \left[-r_{ps}^l a_0^l + r_{ps}^p a_0^p\right]\right|^2\right]^{\frac{1}{2}} \cdot A(r,z) \tag{S34}$$

$$\epsilon_{p,0} = \left(\epsilon_p r_{pp} \cdot \left[1 + m_0 r_0^t a_0^t\right] + \epsilon_s m_0 \left[r_{ps}^l a_0^l + r_{ps}^p a_0^p\right]\right) \cdot \left|\frac{A(r)}{A_0(r)}\right| \tag{S35}$$

$$\epsilon_{s,0} = \left(\epsilon_s r_{ss} + \epsilon_p m_0 \left[-r_{ps}^l a_0^l + r_{ps}^p a_0^p\right]\right) \cdot \left|\frac{A(r)}{A_0(r)}\right|. \tag{S36}$$

For $n \neq 0$:

$$A_n(r) = m_0 \cdot \left(\left|\epsilon_p r_{pp} r_0^t a_n^t + \epsilon_s \left[r_{ps}^l a_n^l + r_{ps}^p a_n^p\right]\right|^2 + |\epsilon_p|^2 \left|-r_{ps}^l a_n^l + r_{ps}^p a_n^p\right|^2\right)^{1/2} \cdot A(r,z) \tag{S37}$$

$$\epsilon_{p,n} = m_0 \cdot \left(\epsilon_p r_{pp} r_0^t a_n^t + \epsilon_s \left[r_{ps}^l a_n^l + r_{ps}^p a_n^p\right]\right) \cdot \left|\frac{A(r)}{A_n(r)}\right| \tag{S38}$$

$$\epsilon_{s,n} = m_0 \cdot \epsilon_p \left(-r_{ps}^l a_n^l + r_{ps}^p a_n^p\right) \cdot \left|\frac{A(r)}{A_n(r)}\right| \tag{S39}$$

With these correspondences, we have for $n = 0$:

$$|c_0|^2 = \left(|\epsilon_{p,0}|^2 + |\epsilon_{s,0}|^2\right) \cdot \frac{\mathcal{W}_{in}}{g\omega} \tag{S40}$$

and for $n \neq 0$

$$\begin{aligned} |c_n|^2 = |m_0|^2 \cdot \Big(&\left|\epsilon_p r_{pp} r_0^t a_n^t + \epsilon_s \left[r_{ps}^l a_n^l + r_{ps}^p a_n^p\right]\right|^2 + \\ &|\epsilon_p|^2 \left|-r_{ps}^l a_n^l + r_{ps}^p a_n^p\right|^2 \Big) \cdot \frac{\mathcal{W}_{in}}{g\omega} \end{aligned} \tag{S41}$$

The energy given by Eq. S22 reads

$$\begin{aligned} \mathcal{W}_{out} &= g\omega \sum_n |c_n|^2 \\ &= \mathcal{W}_{in} \cdot \Big[\big|\epsilon_p r_{pp} \cdot \left[1 + m_0 r_0^t a_0^t\right] + \\ &\qquad \epsilon_s m_0 \left[r_{ps}^l a_0^l + r_{ps}^p a_0^p\right]\big|^2 + \\ &\qquad \left|\epsilon_s r_{ss} + \epsilon_p m_0 \left[-r_{ps}^l a_0^l + r_{ps}^p a_0^p\right]\right|^2 + \\ &\quad |m_0|^2 \cdot \sum_{n\neq 0} \left|\epsilon_p r_{pp} r_0^t a_n^t + \epsilon_s \left[r_{ps}^l a_n^l + r_{ps}^p a_n^p\right]\right|^2 + \\ &\qquad |\epsilon_p|^2 \left|-r_{ps}^l a_n^l + r_{ps}^p a_n^p\right|^2 \Big] \end{aligned} \tag{S42}$$

which gives Eq. 5 of the main text.

For the OAM, Eq. S27 reads

$$\mathcal{L}_{out} = \frac{\mathcal{W}_{out}}{\hbar\omega} \frac{\sum_n \ell_n |c_n|^2}{\sum_n |c_n|^2} \cdot \hbar = \frac{\mathcal{W}_{out}}{\hbar\omega} \frac{\ell_{in} \sum_n |c_n|^2 + \sum_n n|c_n|^2}{\sum_n |c_n|^2} \cdot \hbar = \frac{\mathcal{W}_{out}}{\hbar\omega} \left(\ell_{in} + \frac{\sum_n n|c_n|^2}{\sum_n |c_n|^2}\right) \cdot \hbar$$

The mean OAM per photon thus reads

$$\begin{aligned} \ell\hbar = \frac{\mathcal{L}_{out}}{\frac{\mathcal{W}_{out}}{\hbar\omega}} &= \ell_{in} \cdot \hbar + \frac{\sum_{n\neq 0} n|c_n|^2}{\sum_n |c_n|^2} \cdot \hbar \\ &= \ell_{in}\hbar + |m_0|^2 \cdot \frac{\mathcal{W}_{in}}{\mathcal{W}_{out}} \cdot \left(\sum_n n \left(\left|\epsilon_p r_{pp} r_0^t a_n^t + \epsilon_s \left(r_{ps}^l a_n^l + r_{ps}^p a_n^p\right)\right|^2 + \left|\epsilon_p \left(-r_{ps}^l a_n^l + r_{ps}^p a_n^p\right)\right|^2\right)\right) \cdot \hbar \end{aligned} \tag{S43}$$

which yields Eq. 6 of the main text.

Finally, for the SAM, Eq. S31 reads

$$\begin{aligned} \mathcal{S}_{out} &= 2\frac{\mathcal{W}_{out}}{\hbar\omega} \cdot \frac{\sum_n |c_n|^2 \mathcal{I}(\epsilon_{p,n}^* \epsilon_{s,n})}{\sum_n |c_n|^2} \cdot \hbar\vec{e}_z \\ &= 2\frac{\mathcal{W}_{out}}{\hbar\omega} \cdot \frac{\mathcal{W}_{in}}{\mathcal{W}_{out}} \cdot \Bigg(\mathcal{I}\left(\left(\epsilon_p r_{pp} \cdot \left[1 + m_0 r_0^t a_0^t\right] + \epsilon_s m_0 \left[r_{ps}^l a_0^l + r_{ps}^p a_0^p\right]\right)^* \cdot \left(\epsilon_s r_{ss} + \epsilon_p m_0 \left[-r_{ps}^l a_0^l + r_{ps}^p a_0^p\right]\right)\right) + \\ &\qquad |m_0|^2 \cdot \sum_{n\neq 0} \mathcal{I}\left[\left(\epsilon_p r_{pp} r_0^t a_n^t + \epsilon_s \left[r_{ps}^l a_n^l + r_{ps}^p a_n^p\right]\right)^* \cdot \epsilon_p \left(-r_{ps}^l a_n^l + r_{ps}^p a_n^p\right)\right] \Bigg) \cdot \hbar\vec{e}_z \end{aligned} \tag{S44}$$

The mean SAM per photon thus reads

$$\begin{aligned} s\hbar &= \frac{\mathcal{S}_{out}}{\frac{\mathcal{W}_{out}}{\hbar\omega}} \\ &= \frac{2\mathcal{W}_{in}}{\mathcal{W}_{out}} \cdot \Bigg(\mathcal{I}\left(\epsilon_p^* \epsilon_s r_{pp}^* r_{ss}\right) + m_0 \mathcal{I}\left[r_{ps}^{l*} a_0^{l*} \cdot \left(|\epsilon_s|^2 r_{ss} + |\epsilon_p|^2 r_{pp}\right) + r_{ps}^{p*} a_0^{p*} \cdot \left(|\epsilon_s|^2 r_{ss} - |\epsilon_p|^2 r_{pp}\right) + r_0^{t*} a_0^{t*} \epsilon_p^* \epsilon_s r_{pp}^* r_{ss}\right] \\ &\qquad + |m_0|^2 \sum_n \mathcal{I}\Big(\left[\epsilon_p r_{pp} r_0^t a_n^t + \epsilon_s \left(r_{ps}^l a_n^l + r_{ps}^p a_n^p\right)\right]^* \cdot \epsilon_p \left(-r_{ps}^l a_n^l + r_{ps}^p a_n^p\right) \Big) \Bigg) \cdot \hbar \end{aligned} \tag{S45}$$

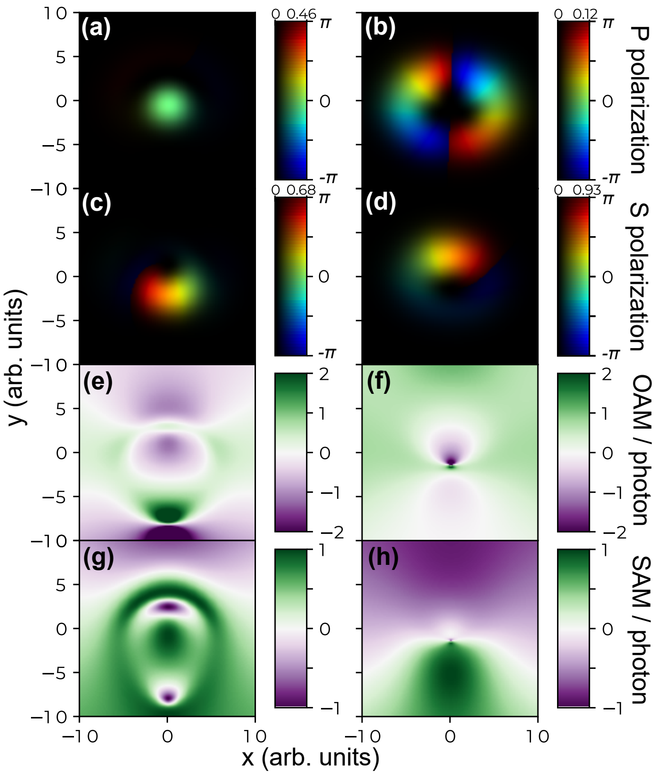

Fig. S1. Analysis of the reflected beam in the far field, for a CL (left column) and CR (right column) incident field with $\ell_{in} = 1$, impinging on a Néel skyrmion. (a,b) p-polarization component. (c,d) s-polarization component. The phase is indicated by the color, and the local intensity corresponds to the brightness of the image. (e,f) OAM density. (g,h) SAM density. In the simulation, the far field is normalized so that the peak of the total intensity is 1.

which gives Eq. 7 of the main text. In Eq. S45 the first term describes a change in SAM given by the Fresnel reflectivity coefficients; the terms proportional to m_0 contribute to the standard longitudinal, polar and transverse MOKE for a uniform magnetization, and as such it is neglected in Eq. 7 of the main text. The terms quadratic in m_0 describe the contribution of non-uniform magnetization.

III. FAR FIELD QUANTITIES FOR A NÉEL SKYRMION

Fig. S1 displays the simulation results for an incident LG mode of OAM $\ell_{in} = 1$, with SAM $s_{in} = \pm 1$, impinging on a Néel skyrmion. It can be directly compared to Fig. 3 of the main text.

IV. DEPENDANCE ON THE INCIDENCE ANGLE

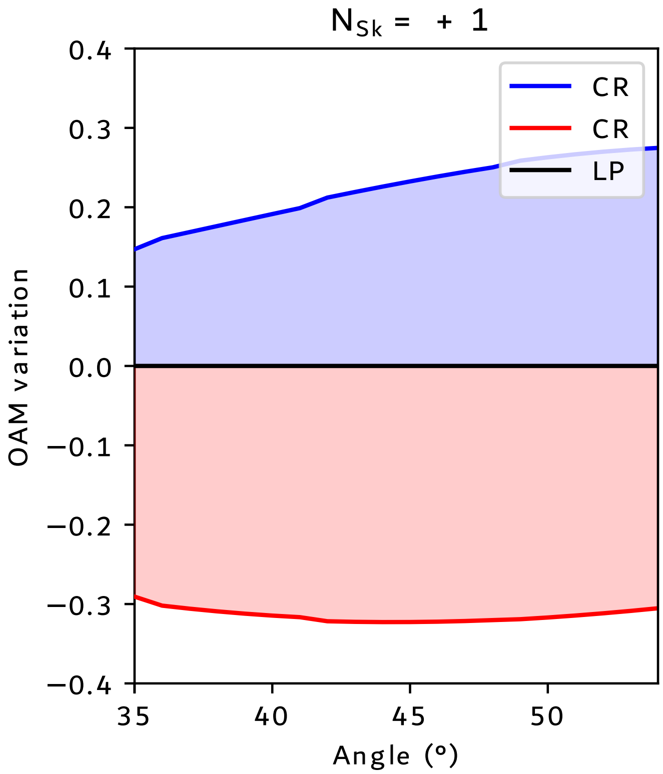

Fig. S2. Variation of OAM upon reflection on a Bloch skyrmion with $N_{\mathrm{Sk}} = 1$ for various input polarizations, with respect to the incidence angle. The photon energy is 711 eV.

[45] C. Piovera, Ph.D. Thesis, Politecnico di Milano (2013).
[53] P. H. Lissberger, Thin film magneto-optics, in Applied Magnetism, Springer Netherlands, Dordrecht, pp. 405–456 (1994).
[54] J. Kuneš, P. M. Oppeneer, H.-Ch. Mertins, F. Schäfers, A. Gaupp, W. Gudat, and P. Novák, Phys. Rev. B 64, 174417 (2001).
[55] C. You and S. Shin, Applied Physics Letters 69, 1315 (1996).
[56] CXRO database, https://henke.lbl.gov/optical_constants/.
[57] K. Y. Bliokh and F. Nori, Physics Reports 592, 1 (2015).
